# Multimodal Hazard Rate for Relapse in Breast Cancer: Quality of Data and Calibration of Computer Simulation

**DOI:** 10.3390/cancers6042343

**Published:** 2014-11-27

**Authors:** Michael Retsky, Romano Demicheli

**Affiliations:** 1 Molecular and Integrative Physical Science, Harvard School of Public Health, Boston, MA 02115, USA; 2 Royal Free and UCL Medical School, Centre for Clinical Science and Technology, University College London, Clerkenwell Building, Archway Campus, Highgate Hill, London N19 5LW, UK; 3 Scientific Directorate, Fondazione IRCCS Istituto Nazionale Tumori, Via Venezian 1, 20133 Milan, Italy; E-Mail: demicheli@istitutotumori.mi.it

**Keywords:** breast cancer, bimodal and multimodal relapse patterns, database quality, early relapses, surgery, perioperative NSAID, transient systemic inflammation

## Abstract

Much has occurred since our 2010 report in *Cancers.* In the past few years we published several extensive reviews of our research so a brief review is all that will be provided here. We proposed in the earlier reports that most relapses in breast cancer occur within 5 years of surgery and seem to be associated with some unspecified manner of surgery-induced metastatic initiation. These events can be identified in relapse data and are correlated with clinical data. In the last few years an unexpected mechanism has become apparent. Retrospective analysis of relapse events by a Brussels anesthesiology group reported that a perioperative NSAID analgesic seems to reduce early relapses five-fold. We then proposed that primary surgery produces a transient period of systemic inflammation. This has now been identified by inflammatory markers in serum post mastectomy. That could explain the early relapses. It is possible that an inexpensive and non-toxic NSAID can reduce breast cancer relapses significantly. We want to take this opportunity to discuss database quality issues and our relapse hazard data in some detail. We also present a demonstration that the computer simulation can be calibrated with Adjuvant-on-line, an often used clinical tool for prognosis in breast cancer.

## 1. Introductory Comments on Quality of Breast Cancer Databases

Researchers who study relapse rates in breast cancer are aware that breast cancer databases often have accuracy problems especially after the first few years of follow-up. There are many reasons for this. The disease runs its course in over 15 years [1,2]. In that time, patients move and modify treatments, data base managers die, retire or change jobs, physicians die or retire, computer systems fail or are changed—just to name the obvious reasons. In Italy patients are known to be generally compliant with physician directives and move infrequently, so few patients there are lost to follow-up. The Milan data have been under the personal control of P. Valagussa, who looked after databases from important randomized clinical trials (e.g., the seminal trial on adjuvant CMF for node-positive early breast cancer) since the first patients were treated decades ago [3]. Valagussa is a no-nonsense person and very diligent. She was doing the data management and would be the first person to discover a problem and she would take action immediately. She does not have a PhD or is a physician but she has spoken on breast cancer to an audience of several thousand medical oncologists and clearly knows much about the disease. Her data are universally considered reputable and our experience substantiates that. We analyzed the Milan data, consisting of 1173 patients undergoing mastectomy without any other adjuvant treatment, with high confidence of their quality and accuracy.

In particular, it should be emphasized that in this database the time of recurrence was determined with high accuracy. Indeed, the proper determination of the time to event is a basic requisite to avoid biases in uncovering the shape of hazard rate curve. In the Milan database, for each patient the time to recurrence was discussed by the clinical team: when recurrence was not detected due to symptoms inducing the patient to anticipate the planned clinical control (about 80% of cases), a review of both clinical and instrumental data was performed.

This is not a trivial matter. To put quality of breast cancer databases in perspective, we can describe some real problems that we personally have encountered regarding such databases. One of us (MR) was Visiting Professor in Department of Medical Oncology at University of Texas Health Science Center—San Antonio (UTHSC-SA) in 1989. This was in the department of the late William McGuire who was an early recognizer of the importance of estrogen and progesterone receptors in breast cancer [4]. MR’s project was to add estrogen receptor to a previously developed computer simulation using the San Antonio data base. This was a very large database consisting of medical records and frozen tissue and serum from 57,000 breast cancer patients. Access was provided to a Cray supercomputer in Austin, TX for this project. The UTHSC-SA staff did not want to provide a full copy of the entire database to a visiting scientist so a few key columns were downloaded and given to MR. The first step was to make sure the existing simulation was consistent with these data. We had technical problems making the simulation run on the supercomputer but with some effort these were eventually solved. Running the Cray with thousands of cases took several hours of CPU time. A serious problem occurred however when we determined that the existing simulation that worked relatively accurately with our own data with 2%–3% error was producing an unacceptable 10%–20% error with the UT data. This was a very complicated hardware/software procedure and changing computer systems especially was a focus of our investigation. We examined and reexamined everything and could not find anything wrong. The problem remained. A few months later we discovered that the columns given to us were mislabeled. Instead of what we thought was date of relapse, we were actually given date of last office visit. With the proper labeling we got nearly our expected accuracy but too much time had elapsed to do the simulation of estrogen.

While the investigation of why the errors were so high was underway, we got a backdoor look into the UTHSC-SA data management process. With such a large database to manage, about 15 young women sat in a row in a long lowly lit room each with a computer and telephone. They knew how important the project was and were intent on doing it well. We noticed a number of sticky notes on the wall in front of each person to remind them to contact such and such doctor’s office to get updates on individual patients. We also found out that these women were not highly paid. They were mostly spouses of US Army personnel stationed at the nearby Ft. Sam Houston. These soldiers were frequently reassigned to other bases so there was a steady turnover of women updating the UTHSC-SA database. It impressed us that with constant turnover of data entry personnel and management partly by sticky notes, it was possible for errors to slip in. The frozen tissue and fluids were stored in freezers in a basement and damaged by a flood in Louisiana a few years ago, but the UTHSC-SA database played an important and historic role in establishing HER-2/neu as a major subject in breast cancer [5].

Having this personal knowledge of what can go wrong even under the best of intentions in large database management, we appreciated the value of one competent person managing a relatively small database in Milan, where data were obtained by physicians performing follow-up visits and not by telephone. Computer simulation is mentally demanding and the researcher must have it lodged in his or her mind that these data are valid. You simply cannot do a good job of developing detailed and error free software to model human cancer growth when doubts exist of the data to be modeled.

## 2. Results and Discussion

Based on computer simulation of the Milan relapse data in 1997 we proposed that something happened at or about the time of surgery to initiate the early wave of relapses within 3–4 years of surgery [6,7]. There are other ways to represent relapse data but it is customary to measure time from primary surgery. This has the effect of allowing us to clearly see events that are synchronized to the surgical event. The early relapse hazard data in Milan data were too sharply peaked to be explained by any purely stochastic process. Something must have happened at surgery to synchronously kick-off metastatic activity for a significant number of patients. We could identify clusters of metastatic relapses that we attributed to single cells that started to divide at the time of surgery and avascular micrometastases that underwent an angiogenic switch at the time of surgery. The latter were most prominent for premenopausal patients with positive lymph nodes. We had a few theories but nothing stood out as overly compelling. As reported in the 2010 *Cancers* paper [1], we could explain much clinical behavior with the surgery-accelerated metastatic growth hypothesis. These include why adjuvant chemotherapy is most effective for premenopausal node positive patients and why mammography works better for women age 50–59 than it does for women age 40–49.

Most relapses in untreated breast cancer are in the early category. The late relapses we proposed to be stochastically driven events that are not synchronized to surgery other than cutting off the supply of cancer cells shed from the primary.

Shortly after the 2010 *Cancers* paper was published, a report was presented by an anesthesiology group from Brussels including P. Forget and M. De Kock [8]. They retrospectively reviewed the outcome from 327 consecutive breast cancer patients treated at their hospital. Patients were given mastectomy performed by one surgeon and this was followed by conventional adjuvant therapy according to accepted consensus reports. The relapse outcome was presented grouped by what drug was used as analgesia. The surprising development was that one analgesic drug resulted in 5-fold lower relapse hazard 9–18 months post-surgery compared to all the other drugs. The best drug was ketorolac which was the only NSAID out of the six in the group.

We were perhaps primed to consider this development. In 2005 we were asked to review a case report from Lebanon in which a 43 year old male with a smoking history was diagnosed with inoperable stage IIIA or IIIB poorly differentiated non-small cell lung cancer [9]. The prognosis for this stage is very poor with 5-year survival of approximately 10%. Treatment was chemotherapy and radiation. While relapse was inevitable, the patient was seemingly doing well 15 months after primary treatment. The patient then incurred a minor trauma to the right temporal skull bone; within a month, the patient reported a rapidly growing 7 cm tumor at the place of the trauma. Imaging revealed tumor had penetrated the skull with meningeal invasion, and while some compression was apparent, it did not yet invade the brain (lung cancer most often relapses to the brain or adrenal glands, but can recur virtually anywhere, including the skeleton). The patient died 15 days later of massive hemoptysis.

The authors suggested that it was not a coincidence that both the growth occurred at the location of the trauma and that the growth started shortly after the trauma. Such a phenomenon has been reported by others [10,11,12]. The mechanisms suggested include some type of trauma-induced angiogenesis of a dormant micrometastasis or dormant malignant cells that happened to be at that place, or perhaps, there were no cancer cells at the site, but circulating cancer cells were entrapped there due to the trauma.

Being that we had a quantitative computer simulation of breast cancer and assuming NSCLC growth was not too much different from breast cancer we could say with confidence that it was not possible that this case report showed surgery induced angiogenesis of some dormant deposit. The only explanation that seemed possible to us was that the minor trauma resulted in inflammation at the site of trauma and that circulating cancer cells were entrapped there similar to what the authors suggested. This was an exaggerated situation to be sure; however in our published comments we suggested that inflammation can be a facilitating precursor to tumor and may be a new and possibly important hematologic metastatic pathway [13].

So it was not too great a leap for us to consider that, since an NSAID at the time of surgery seemed to prevent early relapses according to the Brussels report, this may indicate that surgery somehow resulted in transient systemic inflammation and the inflammation was part of the initiation process for early relapses. We sensed the importance of this, teamed with the anesthesiologists, and analyzed the situation involving perioperative NSAIDs and early relapses and concluded there are a number of biologically sound ways by which the Brussels NSAID intervention prevented transient systemic inflammation following surgery and effectively blocked events leading to early relapse [14,15]. This could explain both the Milan data and the Brussels data. The implications are potentially important since this is an inexpensive and non-toxic treatment, can be done anywhere in developed or developing communities and could (if Brussels data will be confirmed) reduce breast cancer relapses and mortality by 25% to 50%. This is all discussed in our recent reviews.

Demicheli *et al*. have reported similar relapse patterns in other cancers such as NSCLC and Uveal Melanoma [16,17]. Perhaps this is a general effect rather than applying to only a few malignancies. Also Forget, De Kock *et al.* report other breast cancer databases showing this perioperative NSAID effect including breast conservation surgery and they also report a simple preoperative marker that would be related to the systemic inflammation [18,19,20].

### What Other Databases Show a Multimodal Relapse Pattern?

There are several papers reporting on recurrence or mortality that explicitly acknowledge the occurrence of multiple hazard rate peaks [21,22,23,24,25,26,27,28,29,30,31,32,33,34,35], and other papers where such peaks may be seen but are not explicitly acknowledged, although both the peak pattern and its explanations may be different [36,37,38,39,40,41,42,43,44,45,46]. Still another paper reports gene differences between early and late relapses [47]. Regarding the Yakovlev *et al.* paper [27], the main author is now deceased and the document itself does not state anything about multimodal relapse but one of us (MR) spent an afternoon in New York with him discussing this subject. Yakovlev clearly told us that there is a bimodal relapse pattern in his data.

**Figure 1:**
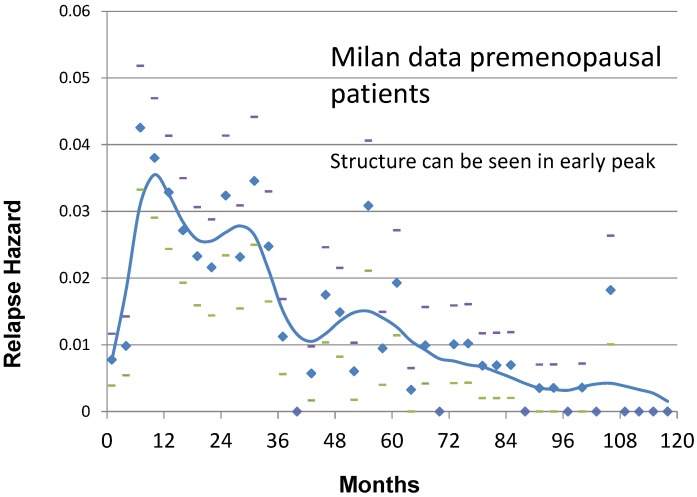
Recurrence hazard rate for Milan premenopausal patients.

About peak patterns, discrepancies are not surprising and may be ascribed to different database quality levels (as previously explained), and different details of the analysis. The discretization of time, for example, is crucial for detecting hazard rate structures: in our experience analyzing events in a 3 month time lag allowed detecting the fine structure of premenopausal recurrence risk during the first three years (Figure 1) that was elusive in the previous 6 month analysis and that is undetectable in a 12 month analysis. Moreover, it is essential to have a clear definition of the analyzed variable, which should be unambiguous (e.g., distant metastasis, death for any cause, *etc.*). In this regard, it is instructive to examine the excellent paper of Karrison *et al.* aimed to evaluate the limit of breast cancer dormancy [25]. The authors “did not see evidence for a second peak in the hazard curve”, an occurrence that may be ascribed to the event they analyzed (“first recurrence or death from breast cancer”). Indeed, while the events “first recurrence” and “death from breast cancer” reliably identify patients who are not cured, the combined event is not a useful end point for study of the time-dependent structure of the hazard of each of the two events. This point is supported by the fact that when the yearly discrete hazard for time to death from all causes for breast cancer patients from the Karrison *et al.* study (1547 patients) and from the Milan study (1173 patients) were compared, both curves display an initial peak at about 3 to 4 years and a second peak at 8 years with an impressive similarity in values [48].

The naive evaluation of Kaplan-Mayer (KM) curves suggests the occurrence of peaks without other details. Indeed, the confidence on the occurrence of “true” peaks instead of artifacts from data processing relies on their constancy when: (a) different smoothing modalities are utilized (we used both Kernel-like smoothing and more sophisticated models such as for example flexible piecewise exponential regression models) and (b) different databases and different subsets of patients are analyzed.

Referring to Figure 1, these patients were untreated with adjuvant therapy. Trimodal relapse pattern is apparent in this case whereas a bimodal pattern is usually seen for treated patient groups [15]. The peak in hazard at 10 months from surgery is the result of surgery-induced angiogenesis of dormant avascular micrometastases according to the computer simulation. Likewise the 30 month peak is identified as single malignant cells that are induced into division at the time of surgery and then stochastically undergo angiogenesis. The broader peak at 50–60 months according to the simulation represents what may be called natural history relapse events and apparently are not stimulated into growth by surgery. The top of that peak identifies when the benefit of removing the primary tumor that sheds metastatic cells first appears so that may be considered temporally tied to the surgical event. The remaining very small hills and valleys after the 50–60 month peak are apparently noise due to very few events and according to the simulation are not associated with or synchronized to the time of surgery. Are the 10, 30 and 50–60 month peaks real or might they be merely an artifact of the numerical analysis? That is a key question and reasonable to ask. We can say these peaks are routinely identified in our analyses, no matter what methodology is adopted, and the timing of the events is constant and quantitatively consistent with our model.

**Figure 2:**
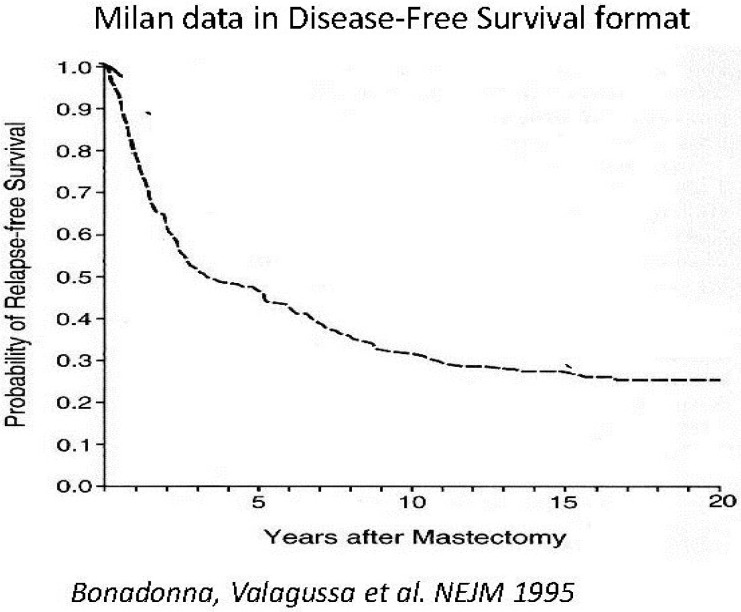
Milan data in disease-free survival format for premenopausal patients. Modified from Bonadonna *et al.* [3].

**Figure 3:**
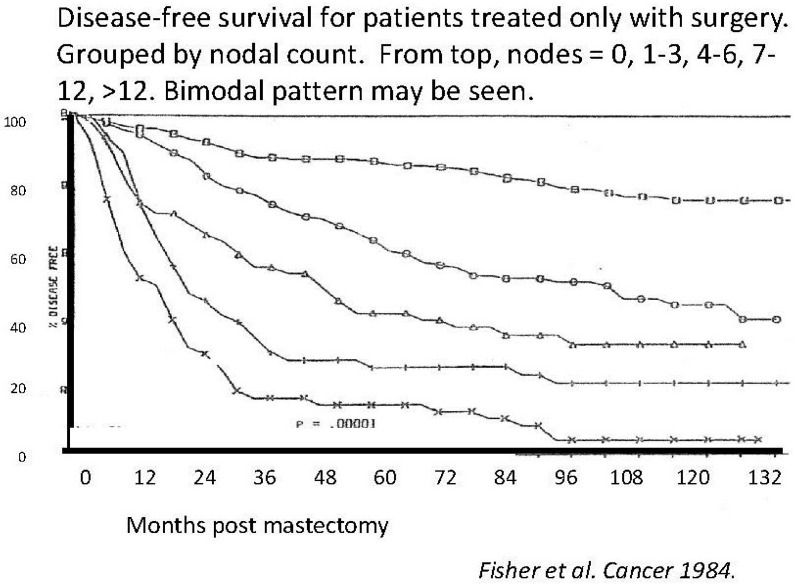
Disease-free-survival reported (modified) from Fisher *et al.* [41].

**Figure 4:**
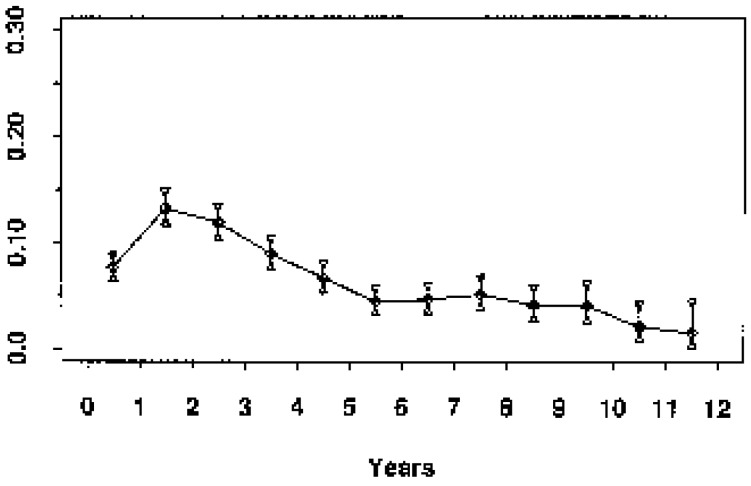
Saphner *et al.* data (Saphner *et al*. ECOG trials, Hazard of Relapse). These data which combine approximately 10 clinical trials for a variety of controls and adjuvant therapies clearly show bimodal hazard. The error bars are small as a result of the large number of patients. Modified from Saphner *et al.* [39]. ECOG is the Eastern Cooperative Oncology Group.

Looking at Figure 2, where the same data as in the prior Figure 1 are presented in the more common disease free survival (DFS) format, it is clear that the plateau between 3 and 5 years represents the end of the early relapse events and the late relapse events are shortly to be seen. Indeed, the hazard rate is related to the slope of the KM curves. With this concept in mind we may look at KM curves and qualitatively detect presence of structured hazard rates, such as in the Fisher *et al*. paper (Figure 3) [41]. This figure has been seen for 30 years but it was only after we identified the multimodal distribution in the Milan data was it clear that Fisher *et al*. data also show a multimodal relapse pattern. Looking at the node negative curve at the top, surgery alone cures 80% of such patients. Of the 20% of patients that relapse, half are in the early wave of 1–3 years post-surgery and the other half are late relapses greater than 5 years post-surgery. For the highest risk category at the bottom, surgery alone is clearly inadequate since all patients relapse and 90% of these are in the early category and 10% in the late category. This is visually and quantitatively what we found for the Milan data. From this figure, the large magnitude of the early relapses is apparent. Over half of all relapses in untreated patients are in the early group. Visually apparent multimodal data from another clinical group are presented in Figure 4.

## 3. Can the Simulation Be Adjusted to Match Specific Clinical Data?

**Figure 5:**
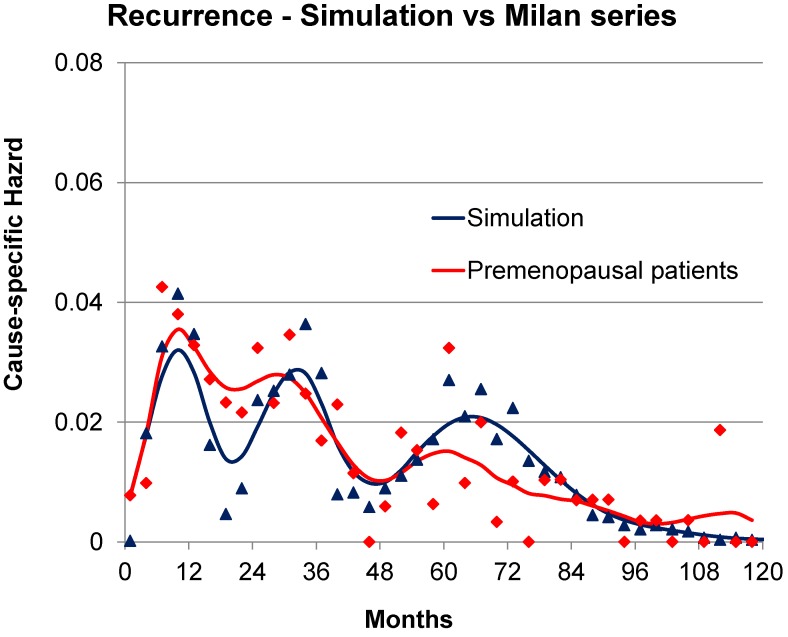
Simulation of Milan data in blue and actual Milan data for premenopausal patients in red. Format is hazard of relapse. We are comparing the Milan data for premenopausal patients and the simulation. We adjusted the simulation to try and agree with hazard for the Milan data. Time scale is 120 months.

**Figure 6:**
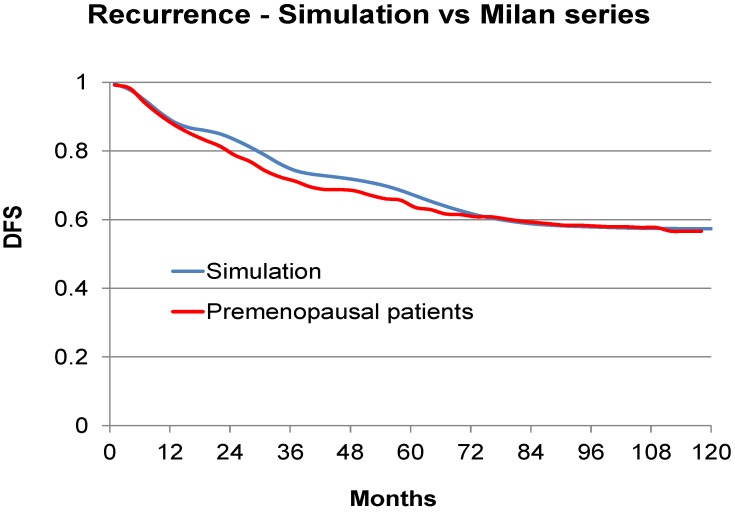
Simulation of Milan data in blue and actual Milan data for premenopausal patients in red. Format is disease free survival.

As an exercise and to demonstrate that the simulation can be adjusted to agree with clinical data, we used the Milan data for premenopausal patients and examined to what exact clinical situation this corresponds. We used the often used and clinically validated website Adjuvant-on-line (http://www.adjuvantonline.com/index.jsp). Milan data were used as an intermediate connection between the simulation and Adjuvant. Milan data are for untreated patients. As Adjuvant provides only 10 year disease-free-survival, it could correspond to different sets of prognostic factors. For the Milan premenopausal data, the corresponding Adjuvant situation could be for a patient 45 years of age, tumor size 2.1 to 3.0 cm, N-, ER unknown, Grading unknown. It also could be for a patient 45 years of age, tumor size 1.1 to 2.0, N+ 1–3, ER unknown, Grading unknown. Hazard of relapse for these data and simulations are shown in Figure 5 and corresponding DFS data are shown in Figure 6.

In Figure 5 we are comparing the Milan data for premenopausal patients and the simulation. We adjusted the simulation to try and agree with hazard and DFS for the Milan data. The cohort that was generated using the simulation consisted of a linear combination of patient groups that were predisposed to relapse at approximately 10 or 30 months after surgery as a result of surgery induced activity plus a group that would relapse with stochastic events only. No other adjustments were made to produce Figure 5 and Figure 6. This calibration exercise was conducted on a trial and error basis with much data traffic between US and Italy where the simulation and analysis software respectively reside. Initial figures were far apart and, as adjustments were made, the simulation and Milan data became more similar. Correspondence is good although slightly less than perfect.

## 4. Discussion—Analysis and Synthesis

We two authors each have backgrounds in physics in addition to doing cancer research. Physicists have long considered *analysis and synthesis* to be a very useful strategy to help understand a complex item. By taking the item apart, examining the individual components and demonstrating how they work together by reassembling the pieces, the scientists demonstrate some new level of understanding. That is precisely what we have done in this breast cancer study. We found two early and one late relapse modes. We have quantified their separate growths in a computer simulation and shown the temporal connection to the surgery event. We show in Figure 5 and Figure 6 that the three relapse modes together can visually resemble clinical data from the Milan database and also agree with Adjuvant-on-line specific clinical situations.

Physicists also ascribe to the scientific method that starts by proposing a testable hypothesis to explain data. An important step here is to attempt to correlate this hypothesis with other observations that have not previously been connected. This has been done as we reported in *Cancers* in 2010 among which is the ability to explain why adjuvant chemotherapy works best by far for premenopausal node positive patients and also why early detection of breast cancer is more effective for women age 50–59 years than it is for women age 40–49 years. We eagerly await the opportunity to prospectively confirm a key prediction that a perioperative NSAID can dramatically reduce early relapses at low cost and low toxicity. One such trial is scheduled to begin this year in Seoul, South Korea at the Samsung Medical Center.

## 5. Conclusions

We have verified that at least in this application a relatively small database consisting of good quality data is far more important than one of large size and questionable quality data. We compliment and thank Pinuccia Valagussa for developing and providing the Milan database upon which all this is based.
